# Lutein-fortified infant formula fed to healthy term infants: evaluation of growth effects and safety

**DOI:** 10.1186/1475-2891-9-22

**Published:** 2010-05-21

**Authors:** Rosario Capeding, Connie P Gepanayao, Nerrisa Calimon, Jowena Lebumfacil, Anne M Davis, Nicole Stouffer, Bruce J Harris

**Affiliations:** 1 Asian Hospital and Medical Center, Medical Office Building, Civic Drive, Filinvest, Alabang, Muntinlupa City, Philippines 1781; 2 Pulo Health Center, Cabuyao, Laguna, Philippines; 3 Wyeth Philippines, 2236 Chino Roces Avenue, Makati City, Philippines 1200; 4 Martek, Baltimore, MD, USA; 5 Stouffer & Associates, 8 Wellesley Way, Medford, NJ 08055, USA; 6 Pfizer (formerly Wyeth) Nutrition, 200 Campus Drive, Collegeville, PA 19426, USA

## Abstract

**Background/Objectives:**

Breast milk contains lutein derived from the mother's diet. This carotenoid is currently not added to infant formula, which has a small and variable lutein content from innate ingredients. This study was conducted to compare the growth of infants fed lutein-fortified infant formula with that of infants fed infant formula without lutein fortification.

**Subjects/Methods:**

This 16-week study was prospective, randomized, controlled, and double-blind with parallel groups of healthy term infants fed either control formula (Wyeth S-26 Gold, designated as Gold) or experimental formula (Wyeth S-26 Gold fortified with lutein at 200 mcg/l, designated as Gold + Lutein). Two hundred thirty-two (232) infants ≤ 14 days postnatal age were randomized and 220 (94.8%) completed the study. Weight (g), head circumference (cm), and length (cm) were measured at Weeks 4, 8, 12, and 16. The primary endpoint was weight gain (g/day) from baseline to Week 16. Safety was assessed through monitoring of study events (SEs) throughout the study and evaluation of selected blood chemistry tests performed at Week 16.

**Results:**

Infants in both treatment groups demonstrated appropriate growth. No differences between treatment groups were found in any of the measures of growth at any of the measurement time points. Both study formulas were well tolerated. The mean values of all measured blood chemistry parameters fell within the modified normal ranges for infants, and the values for both groups for any measured parameter were similar.

**Conclusions:**

Infants fed lutein-fortified S-26 Gold demonstrated growth equivalent to that of infants fed unfortified lutein formula.

## Introduction

Lutein and zeaxanthin are xanthophylls in the family of carotenoids found in common foods including spinach, peas, and broccoli. These compounds are unique being highly concentrated in the macular region of the retina and function as antioxidants and as filters for high-energy blue light [1]. Studies in primates and in adults suggest that lutein and zeaxanthin may help provide protection against oxidative and "blue light" damage [2,3]. Lutein is a structural component of the eye and is a potent antioxidant. Lutein is well suited for protecting the retina from oxidative damage compared with other chain-breaking antioxidants in the eye like alpha-tocopherol (Vitamin E). Lutein can return singlet oxygen to ground state by temporarily becoming triplet-state lutein and then dissipating the energy as heat. This process can be repeated over and over again, because the lutein molecule remains intact after the energy transfer [4]. No data currently exists which demonstrates that lutein supplementation can influence visual acuity in infants, though some studies in adults with visual disorders have shown modest benefits [5,6].

Humans cannot synthesize these carotenoids, therefore blood and tissue levels depend on dietary consumption. Breast milk being the reference standard for infant formula composition, contains lutein and zeaxanthin from the mother's diet, though lutein appears to be the predominant one of the 2 carotenoids [7,8]. The levels of lutein vary widely in breast milk. A 9-country survey conducted on breast milk carotenoid composition among 471 women served as a guide to determining appropriate lutein supplementation levels [7]. The overall mean ± SD from this survey for breast milk lutein plus zeaxanthin was 25 ± 19 mcg/l, but individual country means varied from a low of 15 ± 5 mcg/l in the U.S. to a high of 44 ± 18 mcg/l in Japan. The highest individual lutein concentration measured was 232 mcg/l in China and the lowest was 3 mcg/l in the U.K. These carotenoids are currently not added to infant formula, which has a small and variable innate amount of lutein.

The primary objective of this clinical trial was to compare the growth of healthy term infants fed either Wyeth S-26 Gold (designated as Gold), an infant formula currently marketed by Wyeth Nutrition, or Wyeth S-26 Gold fortified with lutein at 200 mcg/l (designated as Gold + Lutein) for 16 weeks.

The lutein used in fortification of the formula was derived from the marigold flower (*Tagetes erecta L)*. The raw material used was Lutein 20% liquid in Safflower Oil sourced from Kemin Health L.C. (Des Moines, Iowa, USA). This source of lutein also contains zeaxanthin in a ratio of 13:1, lutein:zeaxanthin, and has been determined by the WHO/FAO/Codex Joint Evaluation Committee on Food Additives (JECFA) to be safe for use as a nutrient fortification with an Allowable Daily Intake (ADI) of 0-2 mg/kg [9].

The lutein-fortification level of 200 mcg/l corresponds to the high end of the range of observed breast milk values and potentially could maximize functional effects to infants without any safety risk.

## Materials/Subjects and methods

The study was prospective, randomized, controlled, double-blind with parallel groups of healthy term infants. The trial was conducted between 07-Nov-2005 and 03-May-2006 at 2 study centers: Asian Hospital Medical Center in Muntinlupa City and Pula Health Center in Cabuyao Laguna, Philippines. The Institutional Review Boards/Institutional Ethics Committees (IRB/IEC) of the participating centers approved the study protocol. Infants were randomized into one of two formula groups: Gold or Gold + Lutein, if they met the study's inclusion/exclusion criteria and their parent(s)/legal guardian(s) had made a decision to formula feed their infant prior to study screening and had signed the IRB/IEC-approved informed consent.

Anthropometry data collection procedures were adapted from the Department of Health and Human Services 2000a. This involved the use of two study-associated examiners for each infant.

### Study population

Two hundred forty (240) healthy full term Asian infants ≤ 14 days of age were screened and enrolled. Of these enrolled infants, 232 were randomized to one of the 2 formula groups. One hundred sixteen (116) infants were randomized to each formula group with 110 infants in each group completing the study. Of the 116 infants randomized to each formula group, one infant in each group never received formula at the request of the parent(s)/legal guardian(s) (see Table 1). Infants could be removed from the study at any time at the request of the parent(s)/legal guardian(s), sponsor, or investigator due to formula intolerance, administration of prohibited medications/therapies, and noncompliance with the study protocol. Prior to enrollment into the study infants were fed in accordance with maternal choice and hospital practices. There were no infants who consumed a prohibited therapy or non-study feed during the study.

**Table 1:** Summary of Infant Demography, Baseline Characteristics, and Disposition

		Gold	Gold + Lutein	Total
		(n = 115)	(n = 115)	(n = 230)
**Age **(days)				
	n	115	115	230
	mean ± SD	9.5 ± 3.42	10.0 ± 3.49	9.8 ± 3.46
	min - max	1 - 14	2 - 14	1 - 14
**Gender**				
	Female (%)/Male (%)	60(52)/55(48)	58(50)/57(50)	118(51)/112(49)
				
Weight	Mean ± SD	3169.1 ± 306	3216.6 ± 355	3193 ± 331
				
**Study Disposition (%) **^1^				
Infants Screened				240
Infants randomized^2^		116	116	232
Completed the Study		110 (95)	110 (95)	220 (95)
Discontinued the Study		6 (5)	6 (5)	12 (5)
Reason for study discontinuation (%)				
Adverse Event		4 (3)	3 (3)	7 (3)
Parent/Legal Guardian Request		2 (2)	3 (3)	5 (2)

Population: Intention to Treat

n = Number of randomized subjects who received study formula.

^1 ^The denominator used to calculate percent for study demographics is the number of subjects who received study formula.

^1 ^The denominator used to calculate percent for study disposition entries is the number of subjects randomized.

^2 ^Two subjects were randomized (one in each formula group) but did not receive study formula.

### Study feedings

The study formulas [Gold (control formula) and Gold + Lutein (experimental formula)] were supplied as ready-to-feed liquid, in 250-mL tetrabriks. The 4-month formula supply for each infant was labeled with a unique package number to mask the identity of the formulas. Each study infant was assigned one package number upon randomization and enrollment to the study. The 2 study formulas had the same composition of micronutrients and macronutrients with the exception of lutein, which was added at 200 mcg/l to Gold + Lutein.

Study formula was fed ad libitum. Study formula intake was assessed by parents/legal guardians using a formula weighing scale and recording study formula intake during a 3-day period at Weeks 4, 8, and 12.

The Per-Protocol (PP) population consisted of those infants who did not violate the protocol and who completed the study. Infants consuming any amount of study formula were to be included in the Intention-to-Treat (ITT) analysis. Non-study feeds were defined as any feeding other than assigned study formula that contributed more than 315 KJ (75 Kcal)/day to the infant's diet.

### Randomization

A computer-generated-randomization schedule was used to assign study formula, and the schedule was stratified by gender and assignments balanced per block of four. Infants were randomized (1:1) to receive one of the 2 feeding regimens.

### Efficacy and safety parameters

Infants were evaluated at baseline (designated as Week 0, which could encompass the time period from birth through Day 14 of life) and Weeks 4, 8, 12, and 16.

The primary efficacy outcome measure was infant growth as assessed by weight gain, expressed in grams per day, from baseline to Week 16. Other measures of growth including gains in length and head circumference were assessed. Anthropometry data collection was carried out according to protocol-specified methods. Weight was determined to the nearest 0.01 kg. Length was measured using a length board with a fixed headpiece and a moveable foot piece. Head circumference was measured using a non-stretchable flexible measuring tape. Growth of the infants was compared against US Center for Disease Control (CDC) reference data found on http://www.cdc.gov/growthcharts/[10] as well as Philippine reference growth curves [11] for head circumference. These analyses provided z-scores and percentiles for weight-for-age, length-for-age, head-circumference-for-age and weight-for-length. Secondary efficacy parameters evaluated in the study were visual acuity, measured with Teller Acuity cards, and infant temperament. Study findings with respect to these secondary parameters are not presented here, but will be presented in future publications.

Safety of the infants was monitored by documentation of all study events (SEs) that occurred during the study and by analysis of a single blood sample drawn from each infant at Week 16 and analyzed for albumin, alkaline phosphatase, total bilirubin, blood urea nitrogen (BUN), calcium, creatinine, glucose, phosphorus, and total protein. A single blood sample was chosen to minimize the number of venipunctures for each infant and only serum chemistries were assessed to minimize the amount of blood withdrawn from each infant. Comparisons were made between treatment groups and also with the normal ranges for each of these parameters as proposed by Soldin et al. [12] for a pediatric population. The values from Soldin et al. were used, thus slightly modifying the ranges proposed by Quintiles, the study central laboratory, because this modified range was considered more representative of an infant population. This modified range was reviewed and approved by the principal investigator (PI). SEs were tabulated by preferred term, body system (system organ class), relationship to feeding formula as assessed exclusively by the PI, and outcome of the SE. Criteria for causality assessments of SEs as well as the definition of SEs were detailed in the protocol. Each infant received a complete medical examination at baseline and at Week 16, including a routine fundoscopic examination.

The PI was responsible for complying with the protocol and adherence to GCP/ICH guidelines. A Wyeth study monitor visited the investigator prior to the start of the study and at regular intervals thereafter. All information was recorded on source documents and data were recorded in the case report form screens. Computerized and manual edit checks were performed on all entered data to ensure the data were logical and consistent.

### Statistical analysis

The sample size was determined to have sufficiently large power to exhibit that growth of the Gold group is equivalent to the Gold + Lutein group as measured by average weight gain per day (g/day) measured at Week 16. The two formula-fed groups are equivalent if the true difference between the means is less than 3 g/day.

To determine equivalent growth (weight gain in g/day) between the Gold and Gold + Lutein group, a one-sided (alpha = 0.05) test for equivalence has a 90% power to detect a 3 g/day difference in weight with 42 infants in each treatment/gender group, given a standard deviation of 5.3 as estimated in a previous study [13]. Based on previously conducted trials, it was assumed that many infants might drop out; therefore, a total sample size of 232 infants was randomized to ensure that the minimum number of evaluable infants was at least 186.

The primary endpoint, weight gain (g/day) at Week 16, was analyzed by using an analysis of covariance (ANCOVA). The model includes terms for treatment, gender, baseline age (in days), age at the Week 16 measurement, and baseline weight-for-length z-scores.

A pairwise comparison between the Gold group and the Gold + Lutein group was conducted by examining the difference in least squares means and the associated 90% confidence interval (CI). If the 90% CI of the difference in least squares means was within a difference of -3 to 3 g/day, then the two treatments were determined to be equivalent.

The number of infants with a valid Week 16 weight measurement is the same for the Intention-to-Treat (ITT) population and the Per-Protocol (PP) population; therefore, the results of the primary analysis are the same for the two analysis populations.

Descriptive statistics (n, mean, standard deviation, minimum, median, maximum and 90% CI) were provided for weight, length, head circumference, weight-for-age z-scores, weight-for-length z-scores, length-for-age z-scores, and head circumference-for-age z-scores for each visit for each formula-fed and gender group. Z-scores were calculated using the SAS program offered by the CDC found on: http://www.cdc.gov/nccdphp/dnpa/growthcharts/resources/sas.htm[14].

## Results

Demographics, baseline characteristics, and disposition of study infants are found in Table 1. The study groups were well matched with respect to gender (Table 1) and with respect to maternal age, parity, birth order of the infant (Table 2), and maternal health and socio-economic characteristics (data not shown).

**Table 2:** Summary of Maternal Demography and Baseline Characteristics

		Gold	Gold + Lutein	Total
		(n = 115)	(n = 115)	(n = 230)
**Maternal age (years)**				
	n	115	115	230
	mean	27.3	28.0	27.6
	std	5.96	5.54	5.76
	median	26.0	27.0	27.0
	min	18	18	18
	max	45	40	45
**Parity**				
	1	53(46)	48(42)	101(44)
	2	27(24)	28(24)	55(24)
	3	23(20)	16(14)	39(17)
	> = 4	12(10)	23(20)	35(15)

Population: Intention to Treat

Formula intake was comparable among the two groups. A Week 4, mean intake was virtually identical at approximately 964 mL/day and at Week 12 the Gold group had a mean intake of 1273 mL/day and the Gold + Lutein group consumed 1237 mL/day.

Infant weight gain was used as the primary measure of growth. Using measurements made at baseline and Weeks 4, 8, 12, and 16, the estimated treatment difference of 0.781 g/day in least squares means (90% CI: -0.91, 2.47) was within the interval of (-3 to 3) g/day and the study formulas were considered to support equivalent growth as shown in Table 3.

**Table 3:** Summary and Formula Comparisons of Weight Gain (g/day) at Week 16

	Gold	Gold + Lutein	Total
	(n = 110)	(n = 110)	(n = 220)
**Summary Statistics**			
n	110	110	220
mean	28.48	29.04	28.76
std	5.371	5.030	5.199
median	27.78	28.30	28.17
min	16.9	19.6	16.9
max	49.9	47.3	49.9
			
Model Estimate*			
n	110	110	-
LS Mean	28.41	29.19	-
Standard Error	0.455	0.455	-
90% Confidence Interval	(27.23, 29.59)	(28.01, 30.37)	-
			
The Estimated Treatment Difference*			
Gold + Lutein VS.	0.781	-	-
			
90% Confidence Interval for the Estimated Treatment Difference*			
Gold + Lutein VS.	(-0.91, 2.47)	-	-

Population: Per Protocol = ITT for this parameter

* Estimates created from an analysis of covariance (ANCOVA) with fixed effects treatment, gender, age (in days), age at the Week 16 measurement (in days) and baseline weight-for-length z-score.

The numbers of subjects with valid measurements in the PP (Per-Protocol) and the ITT (Intention-to-Treat) populations are the same.

**Figure 1:**
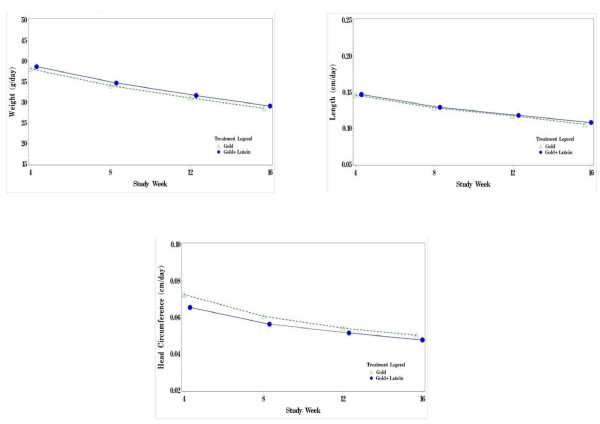
**Mean Velocity Over Time by Each Treatment - Weight (g/day), Length (cm/day), and Head Circumference (cm/day) Population: Intention to Treat**.

Average daily weight gain between the 2 study groups was assessed at defined time points. As shown in Figure 1, rates of weight gain in the Gold group and the Gold + Lutein group were similar at each assessed time point during the study (Weeks 4, 8, 12, and 16); although, over the course of the 16-week study, there was a predictable slowing in the rate of increase in weight gain. When rates of weight gain were analyzed by gender, the addition of lutein to formula had no effect on weight gain at any of the intervals at which it was measured in either the females or the males (data not shown). The standard deviation of weight gain (g/day) at week16 in the Gold group, Gold + Lutein group, and overall was 5.371, 5.030 and 5.199, respectively.

Increased length was observed at all measurement time points with a 24% increase in the mean length for the Gold group and the Gold + Lutein group through Week 16. The change in the rate of increase in length was the same in both groups at Weeks 4, 8, 12, and 16 shown in Figure 1; although, over the course of the 16-week study, there was a predictable slowing in the rate of increase in length. Data were also analyzed for each gender group. The mean rate of increase in length was no different at any measurement time point between formula groups for either females or males.

Both formula groups showed steady increases in mean head circumference over the course of the study (16% in both groups) as shown in Figure 1. Similar to the slowing in growth rates observed with weight and length during the study, rates of increase in head circumference also slowed over the 16 weeks of the study. The rate of increase in head circumference was no different between study groups at any of the 4 measurement time points. When the data were analyzed by gender, similar changes in total mean head circumference increase and rate of gain in head circumference for the Gold group and the Gold + Lutein group were observed in both males and females.

Filipino infant data were compared with the US CDC growth data and z-scores for weight-for-age, weight-for-length, length-for-age, and head circumference-for-age were calculated using the SAS program offered by the US CDC and found on its website:

http://www.cdc.gov/nccdphp/dnpa/growthcharts/resources/sas.htm[10].

**Figure 2:**
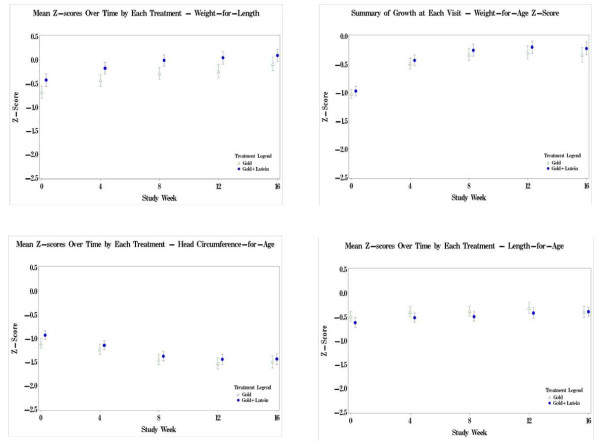
**Mean Z-Scores Over Time by Each Treatment - Weight-for-Age, Weight-for-Length, Length-for-Age, and Head Circumference-for-Age, Population: Intention to Treat.**  Z-scores calculated using the SAS program offered by the US CDC and found on its website: http://www.cdc.gov/nccdphp/dnpa/growthcharts/resources/sas.htm.

**Figure 3:**
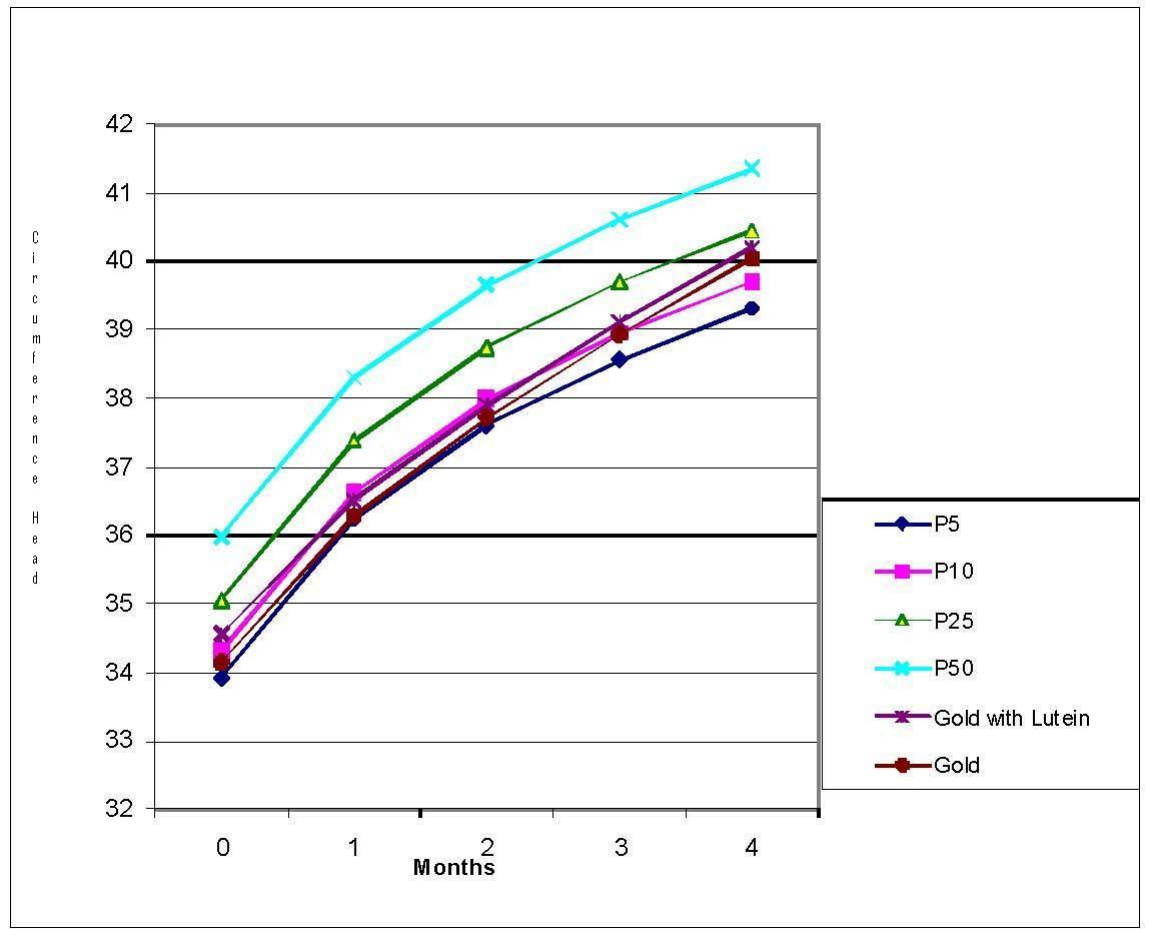
**Head Circumference Male**. Percentile curves generated based on data from the Nutrition Research Institute - Philippine Pediatric Society (FNRI - PPS) Anthropometric Tables and Charts for Filipino Children (see Fiorentino et al., 1992).

**Figure 4:**
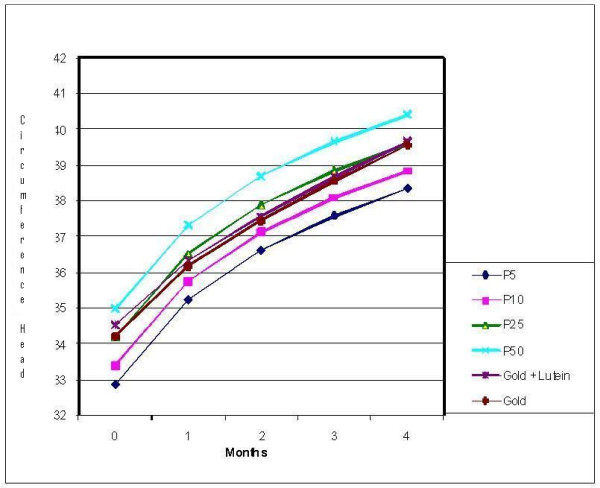
**Head Circumference Female**. * Percentile curves generated based on data from the Nutrition Research Institute - Philippine Pediatric Society (FNRI - PPS) Anthropometric Tables and Charts for Filipino Children (see Fiorentino et al., 1992).

Comparisons using the US CDC reference data are illustrated in Figure 2. Both formula groups had z-scores that paralleled each other for all growth parameters; however a post-study analysis was conducted examining head circumference data from this study compared to a Philippine infant reference population [4]. These Filipino growth charts were based on data from 26,961 Filipino children. When compared with the Filipino growth chart, the head circumference data of this study population followed the growth curve that was established at the time of the baseline measurement, demonstrating age-appropriate growth for this parameter. The head circumferences for the infants track a normal growth rate and are below the 50^th ^percentile which may be the result of a consistent measuring technique with the placement of the tape over this region. This is demonstrated in Figure 3 and Figure 4 for males and females, respectively.

The safety of lutein fortification was also assessed by a comparison of blood chemistries including albumin, alkaline phosphatase, total bilirubin, BUN, calcium, creatinine, glucose, phosphorus, and total protein as well as by a comparison between the two formula groups of frequency and type of clinical SEs that were documented during the study.

Blood samples were obtained from 220 infants, 110 infants from each treatment group. There were no clinically relevant differences in the mean values between the 2 treatment groups for any of the measured parameters. The mean values for each of the parameters were nearly identical between groups and all mean values fell within the range for the modified normal values. Additionally, the fundoscopic exams were normal for both groups.

The mean laboratory values as well as the minimum and maximum laboratory values for each parameter are presented in Table 4. There was comparability in the laboratory values between the two groups and the few values that were outside the normal ranges were not considered to be clinically significant.

**Table 4:** Incidence of Laboratory Normative Values

Test		Gold	Gold + Lutein
		(n = 115)	(n = 115)
ALBUMIN Normal range = 2.1-4.9 g/dL	Mean ± SD Min-Max	4.46 ± 0.25 3.8 - 5.2	4.39 ± 0.22 3.9 - 5.2
			
ALK PHOSPHATASE Normal range = 60-425 U/L	Mean ± SD Min-Max	293 ± 61.8 145 - 474	298 ± 70.4 149 - 640
			
BILIRUBIN TOTAL Normal range = 0-1.0 mg/dL	Mean ± SD Min-Max	0.24 ± 0.07 0.1 - 0.5	0.25 ± 0.08 0.1 - 0.6
			
BLOOD UREA NITROGEN (BUN) Normal range = 1-14 mg/dL	Mean ± SD Min-Max	6.52 ± 1.6 4 - 14	6.39 ± 1.52 3 - 11
			
CALCIUM (mg/dl) Normal range = 7.7-11.5 mg/dL	Mean ± SD Min-Max	10.64 ± 0.37 9.8 - 11.9	10.58 ± 0.34 9.7 - 11.6
			
CREATININE (mg/dl) Normal range = 0.2-0.4 mg/dL	Mean ± SD Min-Max	0.29 ± 0.028 0.2 - 0.3	0.29 ± 0.039 0.1 - 0.4
			
GLUCOSE (mg/dl) Normal range = 57-117 mg/dL	Mean ± SD Min-Max	83.6 ± 8.8 56 - 105	82.9 ± 9.8 45 - 111
			
PHOSPHORUS (mg/dl) Normal range = 3.0-7.5 mg/dL	Mean ± SD Min-Max	6.5 ± 0.46 5 - 7.6	6.43 ± 0.53 4.8 - 9.8
			
PROTEIN TOTAL (g/dl) Normal range = 3.9-7.9 g/dL	Mean ± SD Min-Max	6.58 ± 0.42 5.7 - 7.7	6.43 ± 0.33 5.6 - 7.2

Population: Intention to Treat

Ranges are from Soldin SJ et al. (1999).

Among the 230 infants who consumed any amount of formula, a total of 103 clinical SEs were reported (54 infants in the Gold group and 49 infants in the Gold + Lutein group). There was no clinically relevant difference in the incidence of clinical SEs between the 2 formula groups.

All clinical SEs completely resolved during the study period and, with the exception of the 2 events discussed in the paragraph that follows, were considered mild to moderate by the examining physician.

Two (2) serious SEs were reported for the Gold + Lutein group during the study period, while none were reported in the Gold group. Of the 2 serious SEs, one infant was diagnosed with acute gastroenteritis and the other was diagnosed with bronchopneumonia. Both events were considered by the PI to be unrelated to formula administration. In each case, the infant was hospitalized and the serious SE resolved completely.

## Discussion

The objectives of this study were to assess the effects on growth and safety of healthy term infants fed an infant formula supplemented with lutein. No differences in any of the growth parameters were found between formula groups during the 16-week feeding period.

Data from our study were compared with growth data from a large US infant population. The results of these comparisons were calculated in the form of z-scores and percentiles. For 3 of these 4 parameters (weight-for-age, length-for-age and weight-for-length), the z-scores of the study group means at baseline were less than zero, suggesting the study population was smaller at the outset of the study than a similar population of US infants of the same age. Over the course of the study, the z-score means for weight and length increased to values that were within the second quartile of the US CDC data. These results show that the infants had grown appropriately on both study formulas and achieved growth that was comparable to the mean of the US reference population. The mean head-circumference-for-age data in both study groups were in the same quartile at baseline and at the end of the study.

The frequency and severity of the SEs recorded in the study were similar between treatment groups, were not judged by the PI to be formula related, and in every case the symptoms resolved. The comparability in number of SEs between study formulas supports the conclusion that fortification of an infant formula with lutein is safe for infant consumption. Further support of this conclusion is derived from the blood chemistry data that showed the mean values of all parameters measured fell within the modified normal ranges for infants and that the values between groups for any parameter were no different.

From the data in this study, lutein fortification of S-26 Gold at 200 mcg/L is safe and allows normal infant growth.

## Competing interests

Individuals who conducted this trial were either funded by Pfizer (formerly Wyeth) Nutrition or were employees of Pfizer Nutrition.

## Authors' contributions

BJH and AMD both employees of Pfizer (formerly Wyeth) Nutrition designed the study. RC, CPG, NC, and JL collected the data. NC and JL are employees of Wyeth Philippines. NS (an independent statistician funded by Pfizer (formerly Wyeth Nutrition) and BJH conducted the data analysis and interpretation. BJH wrote the manuscript.

All authors have read and approved the final manuscript.
